# Major Kenneth Mackenzie Cameron

**Published:** 1910-05-28

**Authors:** 


We much regret to announce the death of Major Kenneth Mackenzie Cameron, M.B., R.A.M.C., operating surgeon at the Cambridge Hospital, Aldershot, and late staff surgeon, Army Headquarters, India. Major Cameron, when cycling some weeks ago, was thrown from his machine, sustaining two slight cuts on the knee. Symptoms of tetanus appeared on Tuesday, May 3, and, despite treatment, he died four days later. Major Cameron, who was born in 1868, and received his medical education at Edinburgh, where he graduated M.B., C.M. in 1892, obtained his commission in 1894, and three years later took part in the operations on the North-West Frontier of India. In the South African War he was present at the actions of Rietfontein and Lombard's Kop, and was mentioned in despatches.

